# Clustered DNA Damages induced by 0.5 to 30 eV Electrons

**DOI:** 10.3390/ijms20153749

**Published:** 2019-07-31

**Authors:** Yi Zheng, Léon Sanche

**Affiliations:** 1 State Key Laboratory of Photocatalysis on Energy and Environment, Fuzhou University, Fuzhou 350116, China; 2 Département de Médecine Nucléaire et Radiobiologie et Centre de Recherche Clinique, Faculté de Médecine, Université de Sherbrooke, Sherbrooke, QC J1H 5N4, Canada

**Keywords:** clustered DNA damages, low-energy electrons, transient anions, base modifications

## Abstract

Low-energy electrons (LEEs) of energies ≤30 eV are generated in large quantities by ionizing radiation. These electrons can damage DNA; particularly, they can induce the more detrimental clustered lesions in cells. This type of lesions, which are responsible for a large portion of the genotoxic stress generated by ionizing radiation, is described in the Introduction. The reactions initiated by the collisions of 0.5–30 eV electrons with oligonucleotides, duplex DNA, and DNA bound to chemotherapeutic platinum drugs are explained and reviewed in the subsequent sections. The experimental methods of LEE irradiation and DNA damage analysis are described with an emphasis on the detection of cluster lesions, which are considerably enhanced in DNA–Pt–drug complexes. Based on the energy dependence of damage yields and cross-sections, a mechanism responsible for the clustered lesions can be attributed to the capture of a single electron by the electron affinity of an excited state of a base, leading to the formation of transient anions at 6 and 10 eV. The initial capture is followed by electronic excitation of the base and dissociative attachment—at other DNA sites—of the electron reemitted from the temporary base anion. The mechanism is expected to be universal in the cellular environment and plays an important role in the formation of clustered lesions.

## 1. Introduction 

### 1.1. Clustered DNA Damages and Relationship to Cell Survival

Clustered DNA damages or locally multiple damage sites (LMDS) are defined as two or more lesions that are created by a single radiation track and separated by less than 20 base pairs (bps) (i.e., two helical turns of the DNA molecule). LMDS can consist of pyrimidine and purine lesions (i.e., base damages; BDs), abasic (AP) sites, single-strand breaks (SSBs), and a combination of these lesions. Other than double-strand breaks (DSBs), these combinations can occur in the same or opposite strands [1,2]. 

In damaged cells, the probability of non-repair or the misrepair of a single damaged site is one in 10^9^ to 10^10^ bp [3]. Clustered lesions are orders of magnitude less repairable than those of single lesions, indicating that such damage is much more detrimental to cells. An in vitro study showed that the formation of clustered lesions in the chromosome capping structure can result in the unfolding of existing G-quadruplexes, which can lead to telomere shortening [4]. Bistranded clustered DNA lesions consisting of 8-oxo-7,8-dihydroguanine (8-oxoG), one of the most common oxidative BDs induced by ionizing radiation (IR), results in enhanced mutation frequency, which depends on the location of 8-oxoG in the strand up to ∼20 bp [5]. Bi-stranded clustered lesions are strongly correlated to the structural and dynamic DNA helix distortion [6]. Generally, clustered lesions can lead to mutagenesis, cancer, aging, and neurological disorders in humans [7,8,9].

### 1.2. Clustered Damages Induced by Ionizing Radiation

Nuclear DNA is the most critical cellular target of IR for causing mutations and cell death [10,11]. While chemicals create mainly single or isolated damage sites, the unique characteristic of DNA damages induced by IR is the creation of clustered lesions [1,12,13]. Furthermore, the configuration and spatial distribution of DNA lesions induced by IR are highly relevant to the ensuing detrimental biological effects [14,15,16,17,18]. Considerable studies have shown that LMDS are the characteristic and most toxic lesions induced by all types of IRs [19,20,21,22,23,24]. Readers can refer to historical reviews [1,11,12,25,26,27,28,29].

Monte Carlo (MC) simulation of IR tracks is the typical method for modeling DNA damages [12,30,31]. The molecular damages induced by the radiation of various linear energy transfers (LETs) can be found in a series of studies by Nikjoo et al. [32,33,34,35,36,37]. MC simulations consistently show that clustered damages are characteristic of IR and related to cell death; many different parameters should be considered in the calculations, including those of the track structure of secondary electrons [30,31,38,39,40]. 

DSBs consist of two adjacent SSBs on opposite DNA strands within 20 bps. They are the most well-characterized types of clustered damage [41,42,43,44,45,46], causing mutagenicity and the inhibition of DNA replication [47,48]. γ radiation can induce ~10 to 20 DSBs/Gray, averaging one DSB per 20 to 100 cells [49]. Among clustered DNA lesions, DSBs represent ~20% of the total LMDS [15,16]. 

Base lesions represent another type of DNA damage, which include AP sites (apurinic and apyrimidinic) and a plethora of modifications, such as 8-oxoguanine (8-oxoG), 8-oxoadenine (8-oxoA), thymine glycol, and cytosine glycol [50,51]. The most frequently occurring BDs are the purine AP sites, which are formed either spontaneously by hydrolysis of the C1’-N9 glycosidic linkage of purine nucleotides, or enzymatically as intermediates in the base excision repair pathway. Both AP sites and 8-oxos can be formed in clusters [52,53]. UV radiation can produce clusters of cyclobutane pyrimidine dimers [53]. Base lesions are often involved in non-DSB clustered damages induced by ionizing radiation [25,54,55,56,57]. In X-ray irradiated human cells, non-DSB clustered damage yields are four to eight times higher than those of DSBs [16]. Thus, base lesions are important components of clustered damages [1,4,5,58]. Non-DSB clustered damages can compromise the efficiency of eukaryotic DNA damage repair processes by reducing the ability of glycosylases to excise base lesions, and AP endonucleases to incise the AP sites within a cluster [59]. In general, the ability of cells to repair respective damages depends on the nature of the base lesion. 

The configuration and spatial distribution of DNA lesions induced by IR are highly relevant to the ensuing detrimental biological effects [8,9]. In cells, the reduced repairability of clustered DNA damages could increase the lifetime of the lesions within the clusters, which may cause replication-induced LMDS or mispairing, leading to enhanced mutations and ultimately chromosomal aberrations, genetic instability, and tumor genesis.

### 1.3. Role of LEEs

The interaction of high-energy IR with matters mainly causes ionization, producing immediate positive ions, radicals, and secondary electrons. The latter are the most abundant species produced by IR, such as X-rays, γ-rays, and ions. Approximately 5 × 10^4^ secondary electrons per MeV of deposited energy are generated, with the vast majority having kinetic energies below 30 eV and a most probable energy of around 9–10 eV [60]. Further ionization by a portion of the secondaries produces other generations of low-energy electrons (LEEs). Above the conduction band of biological matter, LEEs are usually considered as “free” electrons, when they do not appreciably perturb the scattering medium. However, at energies of about 15 eV and below, they start polarizing the electronic orbitals of biomolecules, leading to the formation of an electronic polaron [61]. At this point, a polarization cage forms around the electron. When their energy is less than about 3 eV, LEEs start polarizing the phonon modes of water and biomolecules, which deepens that cage, due to the orientation of dipoles and induced dipoles around the negatively charged particle. Energy losses by a LEE eventually lead to what is called the quasi-free state, which becomes the presolvated state with further energy losses. The presolvated state is considered to be an excited state of the solvated electron [61]. Thus, deexcitation of the latter state creates a solvated electron and, unless dissociative electron attachment occurs (DEA) occurs, the LEE becomes the precursor of the solvated electron [61]. In this article, we review cluster damages created by 0.5–30 eV electrons; therefore, it does not include lesions created by quasi-free, presolvated, and solvated electrons. 

Understanding how LEEs damage biomolecules and interact with them is an important topic of investigation in chemical physics that is of relevance to an increasing number of fundamental and applied research areas [61,62,63,64]. When created within the cell nucleus (i.e., close to the genome), LEEs are considered efficient and significant radiation-damage contributors. They can cause SSBs, DSBs, crosslinks, and base modifications in DNA [65,66,67], but it is mostly the unrepaired DNA clustered lesions that are responsible for mutagenic, genotoxic, and other potentially lethal effects in the cell. Therefore, finding the cause of such damages could help understand their destructive action in cancer cells and thus contribute to the development of new improved strategies for cancer treatment with radiotherapy and chemoradiation therapy [68,69].

Today, many techniques exist to investigate the damage induced to biomolecules by LEEs [70]. Simple DNA components (the bases, sugar and phosphate groups, and nucleotides) were investigated in the gas [71,72] or microsolvation phase [73,74], and in the aqueous solution by pulse radiolysis [75,76]. LEE damage induced to multilayer or submonolayer films of DNA segments anchored to a gold substrate or deposited on a conductive substrate was probed by the electron-stimulated desorption (ESD) of ions [77] and XPS [78]. The strand break of biotinylated oligonucleotide origami anchored on Si/SiO_2_ substrates was visualized by AFM with streptavidin [79]. Time-resolved photoelectron spectroscopy followed the dynamics of transient negative ions from an iodide–nucleobase clusters model system, which provided a novel window to study the LEE attachment to nucleobases [80]. For almost two decades, damage products were analysed ex vacuo after bombardement with LEEs of a multilayer film deposited on a metal substrate under ultra-high vacuum (UHV) [65]. New techniques also have been developed to produce LEEs outside UHV, such as for example passing 30-keV electrons from a scanning electron microscope through a 100-nm thick Si_3_N_4_ membrane to generate LEEs (<100 eV) in a DNA solution [81]. Furthermore, with techniques of femtosecond laser filamentation or visible and UV light incidence on various types of plasmonic nanostructures [82,83,84], LEEs can be exclusively produced in solution; the subsequent generation of presolvated and solvated electrons and their reactions can be followed in real time. Each of the methods mentioned in this paragraph have their advantages depending on the nature of the target and the type of information desired.

This article reviews the results obtained so far regarding the production of cluster damages induced in duplex DNA by 0.5 to 30-eV electrons. It is focused mainly on the direct effect of LEEs studied with multilayer film technology in vacuum and under atmospheric pressure. Apart from DSBs, it was only recently that LMDS were investigated by LEE impact. Electron impact under a vacuum of submonolayer and multilayer coverage of substrates by biomolecules is presently the only method that allows for the investigation of the energy dependence of LEE-induced damage in large biomolecules, and thus gains insight into the mechanisms leading to bond scission. For more general reviews of the results of multilayer film experiments, which do not include BDs and related cluster lesions, the reader is referred to previous review articles [65,66,69]. We note that the biological effects of Auger electrons have also been reviewed recently [85]. 

## 2. Experimental Techniques 

### 2.1. Target Preparation and LEE Irradiation

Multilayer films of DNA are prepared by lyophilization [86] or the self-assembly method [87]. Intercalating 1,3-diaminopropane with plasmid DNA onto highly oriented pyrolytic graphite produces more uniform films [87] than lyophilization. For the latter process, target molecules are deposited on the metal substrate, such as Ta. Detailed target preparation by lyophilization and intercalation can be found in references [65,86,87]. The condensed multilayer films are usually bombarded in UHV, with LEEs of energies that can be varied from 0.5 to 100 eV. In UHV, LEEs are produced by an electron gun or a monochromator that has beam resolution varying from 10 to 300 meV [86]. By changing the potential between the substrate (ground) and the center of the filament of the LEE source, the energy of incident electrons can be varied. The absolute electron energy is obtained by measuring the lowest potential for electron transmission through the film, which is considered to correspond to zero eV. The diameter of the electron beam and the working distances from the target can be varied between 2–50 mm and 10 to 50 mm, respectively. 

### 2.2. Analysis of DNA Conformation Variations and Cell Survival

After recovery of the LEE-irradiated samples from vacuum, various configurations of DNA, corresponding to crosslinks, SSBs, DSBs, and supercoils are analyzed by agarose gels electrophoresis stained with a fluorescent dye, such as SYBR Green [86]. The fraction of each DNA form is quantified by image software from their respective band in the gel. Alternatively, fluorescence microscopy, such as γ-H2AX foci staining, can be applied as another effective DSB probe [88]. 

As a means of quantifying the damage to plasmid DNA, it is also possible to directly measure the transformation of cells whose survival depends on intact DNA [89]. In this case, LEE-irradiated plasmid DNA is transferred into *E. Coli* bacteria to increase their resistance against an antibiotic. For example, *E. Coli* bacteria can be incubated in an ampicillin-rich environment that would normally kill them [89]. However, the cells can survive by the injection of undamaged plasmids that encode an enzyme that is capable of inactivating the antibiotic. The correlation of DNA damages with cell survival is determined by the parallel analysis of DNA damages and cell survival. 

### 2.3. Enzyme Treatment for Measuring Base Damages

Base damages are revealed by treating irradiated samples recovered from vacuum with base excision repair (BER) glycosylases [90] and quantifying the differences in the yields of different damages with and without the enzymes. The irradiated DNA is treated with *E. Coli* base excision repair endonuclease III (Nth), formamidopyrimidine N-glycosylase (Fpg), and endonuclease III. The DNA repair enzymes Nth and Fpg can specifically recognize and remove several modified pyrimidines and purines, respectively, by hydrolysis of the glycosidic bond to form strand breaks, which are detectable in the electrophoretic assay described in Section 2.2 [91]. For instance, Fpg can excise 8-oxo-7,8-dihydroguanine and subsequently form apurinic/apyrimidinic (AP) sites by β-elimination, resulting a one-nucleotide gap displayed as a strand break [92]. Nth similarly removes damaged pyrimidines by an N-glycosylase step to produce an abasic site. Since the DNA glycosylase can cleave the N-glycosylic bond between the base lesion on the DNA and the deoxyribose, the free base is released, and the AP site is left, which then produces a DNA strand break by cutting the AP site using an AP endonuclease or a DNA glycosylase. Thus, a base modification can be transformed to a strand break after BER. 

Generally, samples are incubated with the respective enzyme at 37 °C for 0.5 to 1 h to excise specific base lesions and form strand breaks that can be detected by electrophoresis. Parallel experiments are also conducted with DNA untreated with enzymes, but also kept at 37 °C for 60 min, to reveal any heat labile effects. Single molecule fluorescence is applied to observe the lesions linked to DNA glycosylases [93], which insert a wedge amino acid into the DNA helix to probe for alterations in base pairing, base stacking, and sugar puckers produced by the lesion. One sample configuration is 6-his tagged glycosylase conjugated to a streptavidin Q-dot through a biotinylated anti-His antibody [93]. The antibodies of in situ colocalization assays against the human repair enzymes 8-oxoguanine-DNA glycosylase (OGG1) or AP endonuclease (APE1) can also be used to reveal damage foci corresponding to non-DSB base lesions and abasic sites, respectively [92,93]. 

## 3. Clustered DNA Damages Induced by LEEs

### 3.1. DSBs

**Figure 1:**
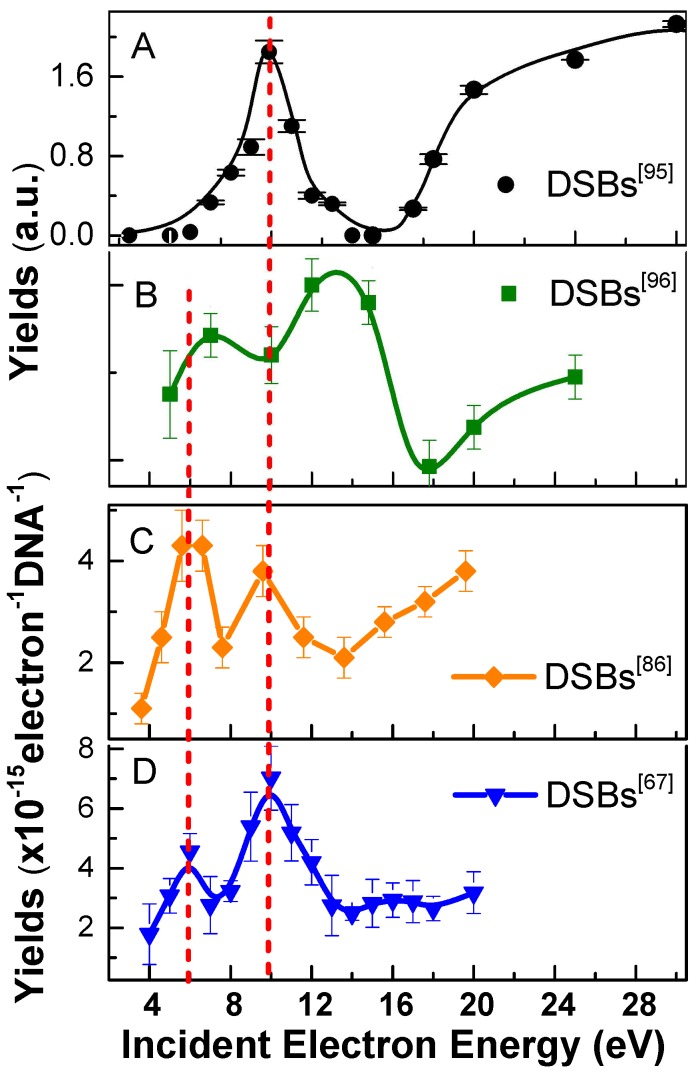
DSBs induced by LEEs below 30 eV in: (**A**) pGEM3Zf(−) plasmid DNA (3197 bps) from ref [95], (**B**) P14 plasmid DNA (6360 bps) from ref [96], (**C**) pGEM3Zf(−) plasmid DNA from ref [86], (**D**) pGEM3Zf(−) plasmid DNA from ref [67]. Dash lines indicate the energies of two prominent peaks around 6 and 10 eV.

So far, DSBs have been the most investigated clustered lesions induced by LEE impact on DNA [67,86,94,95,96]. Figure 1 shows the energy dependence of the yields of DSBs from various film experiments in vacuum [67,86,95,96]. The yields in Figure 1A,C,D were obtained from the initial slopes of exposure–response curves, indicating the probability that a single electron will form a DSB. The result obtained with dry pGEM3Zf(–) plasmid DNA show two maxima around 6 and 10 eV. In those recorded with *hydrated* P14 plasmid DNA (Figure 1B), the two peaks are shifted to higher energy. Such a shift is consistent with the detailed investigation of the effect of water on LEE damage induced to the small oligonucleotide GCAT consisting of the four DNA bases, for which resonance peaks shift to higher energy compared to dry GCAT [97]. The two maxima and the one at 10 eV in the upper curve were ascribed to core-excited resonances arising from the electron capture by the positive electron affinity of electronically excited states of the bases. Decay of such a transient anion (TA) by autoionization can leave the base in an electronically dissociative excited state. If this happens and dissociative attachment of the escaping electron occurs at another site within DNA, the process can lead to a DSB [67]. 

Cai et al. employed photoemission from X-ray irradiated tantalum to produce LEEs [98]. LEEs with an average energy of 5.8 eV and a distribution peaking at 1.4 eV could induce DSBs with G values (i.e., yields per energy deposited in DNA) of 8 to 22 nmol/J, which is 1.6 times larger than that of 1.5-keV photons. Applying a similar and improved technique to produce LEEs, Alizadeh et al. measured conformational damages from dry, oxygenated, and humid films of DNA, LEE-irradiated under standard atmospheric temperature and pressure (SATP) [99]. Their results were compared with the yields produced by 1.5-keV X-rays. In comparison to UHV experiments, the presence of H_2_O led to a higher formation of DSBs with G values of 8 ± 2 and 21 ± 5 for X-ray and LEEs, respectively. The latter corresponds to the indirect and direct effect of LEEs. When water-saturated DNA was exposed to an oxygen atmosphere at SATP, the G value for DSBs induced by LEEs increased by a factor of 31, compared to dry DNA in a nitrogen environment at the same pressure. Furthermore, mixtures of H_2_O and O_2_ increased the formation of DSBs by factors of 11.8 by LEEs in comparison to a dry H_2_O + N_2_ environment, which is almost seven times higher than that from X-ray photons. These indicated that LEEs can induce DNA clustered damages synergistically through the direct and indirect effects of radiation.

### 3.2. Clustered Lesions Involving Base Damage

**Figure 2:**
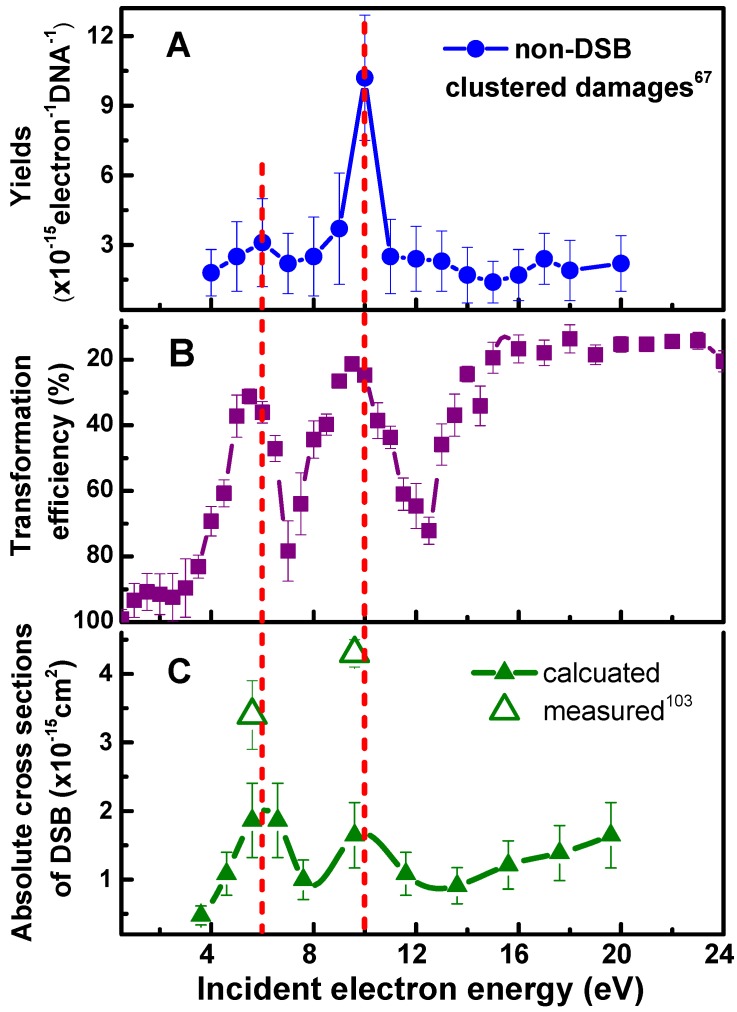
(**A**): Energy dependence of non-DSBs clustered damages [67]. (**B**): Inverted percentage curve that represents the transformation efficiency of *E. coli* cells. It was produced by irradiating with 0.5–24 eV electrons the pGEM3Zf(−) plasmid required for survival of the competent cells. (**C**): Absolute cross sections for inducing DSBs by 4–20 eV electron impact on the pGEM3Zf(−) plasmid [103]. The solid points are calculated from the effective yields with penetration factor *f* = 0.29 ± 0.14 [86]. Dash lines indicate that the energies of the two prominent peaks around 6 and 10 eV of non-DSB clustered damages correlate well with those of cell death.

MC simulations have shown that the simple clustered DNA damage consisting of an SSB and an adjacent BD comprises 90% of the total clustered DNA lesions induced by LEEs below 150 eV [39]. Among the SSB+BD clustered lesions, 80% involve only one BD [39]. Figure 2A shows the yields of non-DSB clustered damages, i.e., multiple interstrand BDs or an SSB with an interstrand BD within 20 bp, as a function of electron energy [67]. The resonances at 6 eV and 10 eV appear at the same energy as those in the DSB yield functions of dry DNA in Figure 1, indicating that non-DSB clustered lesions could be caused by the same initial process that produces DSBs; i.e., the initial captured by a base of the impinging electron leading to the formation of a core-excited resonance. Moreover, the two maxima observed in the cell-deficiency function of LEEs [100] shown in Figure 2B correlate well with those shown in Figure 1 and Figure 2A,C. Curve 2B represents the transformation efficiencies as a function of the energy of LEEs bombarding a plasmid responsible for the survival of the bacteria, as explained in the experimental section. In this manner, the results of curve 2B serve to relate LEE-induced damages to cell death. A detailed analysis of DNA damages induced by 10-eV electrons has also showed that non-DSB clustered lesions are the most likely to be responsible for inactivating the cells [89]. The energy correlation between all the peaks in the curves of Figure 1 and Figure 2A provides further evidence that the LEE-induced clustered lesions that are formed by TA decay into destructive channels, which can be lethal to cells (i.e., they can decrease plasmid DNA functionality, which increases cells death). 

## 4. Cross-Sections of Clustered DNA Damages

Cross-sections (CSs) for LEE-induced clustered DNA damages are important input parameters in MC simulations to generate microscopic absorption doses and quantitate, down to the nanoscopic level, the biological effects of IR [101]. Recently, methods have been developed to obtain absolute CSs for LEE-induced DNA damages in thin-film experiments by applying mathematical survival models to the raw data [102,103,104]. Figure 2C shows the measured absolute CSs of DSB (open points) and the energy dependence (solid points) estimated from effective yields, assuming a constant electron penetration factor (*f*) of 0.29 ± 0.14 for all energies [103]. The measured absolute CSs for DSBs and non-DSB clustered damages induced by 10-eV electrons are 4.3 ± 0.2 and 6.5 ± 1.6 × 10^−15^ cm^2^, respectively [103,105]. In comparison, the CS of DSBs induced by 50-eV electrons were deduced from an effective yield with *f* = 0.45 ± 0.21 to be 2.7 ± 1.5 × 10^−15^ cm^2^ [101]. The CS at 10 eV being larger than that at 50 eV is surprising considering that at 50 eV, the ionization CS is almost maximal. This comparison clearly testifies the ability of LEEs to cause clustered damages via the decay of TAs in the DEA and autoionization channels. Furthermore, according to the results of Alizadeh et al. [99] mentioned previously, it is quite realistic to conjecture that the CSs derived from dry-DNA films in vacuum should be increased by an order of magnitude or more, when LEE interaction with DNA occurs at atmospheric pressure in the oxygenated and hydrated environment of the cell. Furthermore, binding a chemotherapeutic agent to DNA can also increase LEE CSs. For example, when cisplatin is chemically bound to DNA in a ratio of 5:1, the CSs of DSBs and non-DSB clustered damages at 10 eV increase to 9.3 ± 0.4 and 8.2 ± 0.3 × 10^−15^ cm^2^, respectively; i.e., they increase by factors of 2.2 and 1.3 compared to unmodified DNA [105]. 

## 5. Enhancement of Clustered DNA Damages by Chemotherapeutic Agents and Radiosensitizers

Pt-based chemotherapeutic agents (CAs), including cisplatin, oxaliplatin, and carboplatin, are among the most commonly used drugs in clinical concomitant chemoradiation therapy (CRT) [106]. An enhancement factor larger than unity, which is defined as the ratio of the measured damage in the Pt–CA–DNA complex divided by that in non-modified DNA, was consistently observed in previous studies upon LEE irradiation [107,108,109,110,111,112]. Much larger enhancement factors (EFs) of 2.4–3.1 were found for DSBs induced by 10-eV electrons compared to SSBs [108,111]. Although no DSBs were observed in unmodified DNA below 4 eV [110], DSBs could be produced in Pt–CA–DNA complexes by 0.5-eV electrons, with yields similar to those of 1.5-eV X-rays [109]. The study demonstrates that DSBs can be induced by a single-electron hit in Pt–CA–DNA complexes at energies where electrons are abundantly created by IR. Even at 4.6 eV, the yield of DSBs for pure DNA is relatively low, i.e., it is possibly close to the detection limit, which leads to a huge EF for cisplatin–DNA complexes [111]. 

The enhancement of non-DSB clustered damages induced by 10-eV electrons was also observed in Pt–CA–DNA complexes [105,111]. EFs of 1.4–1.7, 1.1, and 1.4 caused by the respective binding of cisplatin, carboplatin, and oxaliplatin to DNA show that the physicochemical radiosensitization mechanism of cisplatin and its analogues could be universal; i.e., the Pt–drug covalently bound to DNA weakens the DNA structure [106], and could favor the initial formation of core-excited resonances, leading to the enhancement of C–O bond scissions (strand breaks) and/or base lesion simultaneously, and hence the formation of clustered lesions. The enhancement of clustered damages in Pt–CA–DNA complexes compared to non-modified DNA is expected to play a significant role in the synergy mechanism of CRT [69,106]. 

Another radiosensitizer causing cluster damages that has been investigated by LEE impact is 5-bromouracil (5BrU) [113]. After the bombardment of T5BrUT with 10-eV electrons, total damage increased by more than 50% compared, under the same conditions, to that in TUT, where U is uracil and T is thymine. Irradiated productsincluded TUT (40%), free U, T, 5BrU (23%) and fragments (13%), which correlated to C–N, C–O, or C–Br bond cleavages, respectively. The multiple products arising from the single-electron hit suggested that 10-eV electrons can induce the cleavage of two bonds in the same DNA strand via electronic excitation followed by DEA [112]. This detailed study of all the products induced by LEE impact on a simple oligonucleotide provided evidence of the formation of clustered damage and suggested that the above mechanism could be applied to the radiosensitization of biological DNA by 5BrU. 

## 6. Mechanism of clustered DNA damage induced by a single LEE

Below 20 eV, DNA damages induced by LEEs essentially involve the formation of two major groups of core-excited resonances at 6 and 10, which are most likely due to electron attachment to a base [67]. The subsequent decay of these TAs into dissociative channels produces not only single bond scissions, but also clustered damage. The question is: how can a single electron of 4 to 12 eV produce two or more close lesions in DNA? Considering the CSs involved [101], the formation of clustered lesions, by two successive collisions within 20 bp, of a single LEE has a much too low probability to account for single-electron generated double lesions with yields only about one order of magnitude lower than those of SSBs [86]. 

**Scheme 1:**
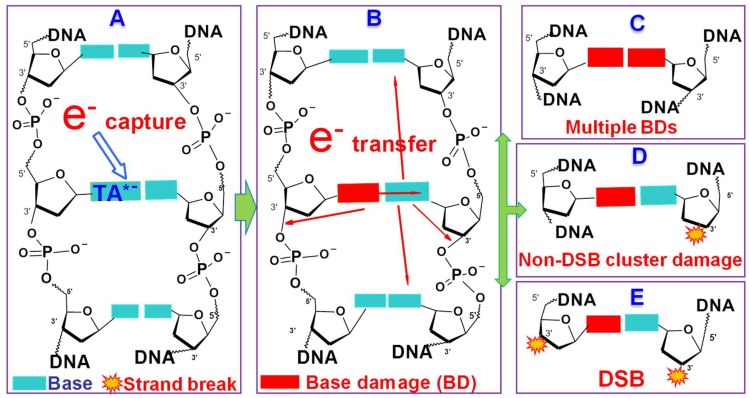
Diagram of the reaction pathways for a single LEE to produce all possible types double-lesion cluster DNA damage. The electron (e-) is initially captured by a base, forming a core-excited transient anion (TA) (**A**). An electron autodetaches from the base anion, leaving the base in a dissociative state (**B**). The base is damaged and the additional electron transfers to other sites where DEA occurs (**B**). Transfer to the opposite base can result in two adjacent BDs (**C**), whereas transfer to the phosphate unit in the same or opposite strand, can cause a strand break via C−O bond breakage (SSB via DEA + BD) (**D**) or a DSB, if the BD on the left strand is converted to a strand break (**E**). Electron hopping between bases can create a BD or SSB farther away from the initial electron capture site (Copyright 2019 American Chemical society [67]).

Scheme 1 illustrates the transfer mechanism recently proposed to explain the formation of all the observed LEE-induced damages in DNA [67]. Using this scheme, we can explain the large yields for the formation of clustered lesions by a single LEE. As shown theoretically [114] and experimentally [86,112,115] up to 10 eV, a LEE interacting with DNA has a high probability of being first captured by a base. To produce cluster damage, the 6-eV and 10-eV core-excited resonances of the bases must first decay by autoionization and leave the base in a dissociative state. This decay mode damages the base. By hydrogen abstraction, the initial base radical can also transform into an SSB within the same strand [116]. If the autoionizing electron transfers to another nearby site, *it can break another bond via DEA*. The latter is the most likely process, since in most cases, the emitted electron does not have enough energy for electronic excitation. Owing to the possibility of electron transfer along the chains, the second damaged site can be located within 20 bp [117]. Hence, electron transfer after dissociation of the initial base can lead to a cluster damage, whose type depends on the autoionizing-electron receptor site (i.e., a base or a phosphate group) and the probability of conversion of a base damage to a strand break [117]. Following autoionization at the initial TA site, the additional damage could be an SSB or BD on the same or opposite strand. The production of the simplest interstrand cluster damages, i.e., a double BDs, SSB with an adjacent BD, and DSBs, are shown in Scheme 1.

## 7. Conclusions

DNA clustered damages are characteristic lesions induced by IR, which are highly relevant to the ensuing detrimental biological effects. LEEs generated in large quantities by IR can efficiently lead to DSB and non-DSB clustered lesions. The energy dependences of the yields of these lesions show two maxima at 6 eV and 10 eV, which are ascribed to core-excited TAs. It has been suggested that these latter produce cluster lesions by their decay into multiple bond cleavage channels via autoionization, followed by electron transfer and DEA. The chemical binding of Pt–CAs to DNA leads to the enhancement of cluster damages, particularly DSBs. The mechanism of clustered damages can be attributed to the capture of a single electron by a base followed by multiple electron transfer pathways. In conclusion, LEEs could play an important role in the formation of clustered damages and constitute an essential element of our understanding of the most deleterious genotoxic stress in radiolysis and radiobiology. Thus, knowledge of LEE mechanisms of damage is crucial to the basic physical chemistry underlying a number of applied fields, including cancer treatment by laser or radiotherapy (with or without chemotherapy) and laser nanosurgery [118].

## Abbreviations

A	adenine
AP	Abasic site (apurinic or apyrimidinic)
BD	base damage
BER	base excision repair
bp	base pair
C	cytosine
CA	chemotherapeutic agent
CRT	chemoradiation therapy
CS	cross section
DEA	dissociative electron attachment
DNA	deoxyribonucleic acid
DSB	double strand break
EF	enhancement factor
*f*	penetration factor
Fpg	formamidopyrimidine N-glycosylase
G	guanine
IR	ionizing radiation
LEE	low-energy electron
LET	linear energy transfer
LMDS	locally multiple damage sites
MC	Monte Carlo
Nth	base excision repair endonuclease III
SSB	single strand break
T	thymine
TA	transient anions
U	uracil
